# Systemic vasculitis diagnosed during the post-partum period: case report and review of the literature

**DOI:** 10.1186/s40748-023-00147-3

**Published:** 2023-02-08

**Authors:** Sophie Demotier, Pauline Orquevaux, Yohan N’Guyen

**Affiliations:** Service de Médecine Interne, Maladies Infectieuses Et Immunologie Clinique. Hôpital Robert Debré. Centre Hospitalier Universitaire Reims, 51100 Reims, France

**Keywords:** Vasculitis, Post-partum, Pregnancy, Delivery

## Abstract

**Introduction:**

The vasculitis diagnosed specifically in the post-partum period are less well known. We report here such a case followed by a descriptive review of the literature.

**Case report:**

A 25 year-old French nurse reported abrupt-onset musculoskeletal pain 15 days after delivery of her first infant. Her first pregnancy was uneventful. The physical examination yielded only bilateral conjunctivitis and purpuric eruption of lower limbs, and complementary investigations evidenced pulmonary renal syndrome in connection with the diagnosis of Granulomatosis with Polyangiitis.

**Methods:**

We screened previous articles in Medline database using keywords (i) “post-partum” or “puerperium” (ii)”peripartum” (iii) “pregnancy” associated with “vasculitis”. Full texts were obtained from case reports or cases series whose title or abstract included keywords of interest (or synonyms). These references were secondarily excluded if the diagnosis of vasculitis was not confirmed or made before or more than 6 months after delivery and if vasculitis occurred only in the new born or affected only the cerebral vasculature or the retina.

**Results:**

Fifty-six cases of vasculitis diagnosed in the post-partum period were included, 50 (89.3%) were secondary to an immunological process and 44 corresponded to primary vasculitis, 4 were secondary to Systemic Lupus Erythematosus, 1 to cryoglobulinaemia and 1 to cryoglobulinaemia associated with inflammatory bowel disease. The main primary vasculitis diagnosed were Takayasu Arteritis (*n* = 10), Eosinophilic granulomatosis with polyangiitis (*n* = 9), Granulomatosis with Polyangiitis (*n* = 7), Periarteritis Nodosa (*n* = 5) and Behcet’s disease (*n* = 4). The first symptom occurred before delivery in 26 (59.1%) and maternal death occurred in 4 (9.1%) out of the 44 primary vasculitis cases.

**Conclusion:**

The vasculitis diagnosed in the post-partum period were mainly primary vasculitis among patients in whom the diagnosis had not been made during pregnancy. In less than half of cases and as described in ours, there were no reported symptoms before delivery. Therefore, the physicians should pay attention to symptoms and keep a high degree of suspicion for vasculitis before as well as after delivery.

## Introduction

Systemic vasculitis represent a wide range of diseases that can be either primary as well as secondary to other processes such as drug exposure, connective tissue disease or infection [1]. Despite several changes during the last years [1], the nomenclature of non-infectious vasculitis is now based on the Chapel Hill classification [2].

While previously discouraged, pregnancy is now described among women suffering from non-infectious systemic vasculitis [3]. The outcome of such pregnancies is reported to be worst when vasculitis was active before pregnancy or diagnosed during pregnancy [3, 4].

However, there are less data concerning systemic vasculitis diagnosed specifically in the post-partum period. We describe here a case of primary systemic vasculitis diagnosed in the post-partum period, without any symptoms during pregnancy, before we make a descriptive review of the literature focusing on vasculitis diagnosed specifically in the post-partum period.

## Case report

**Fig. 1:**
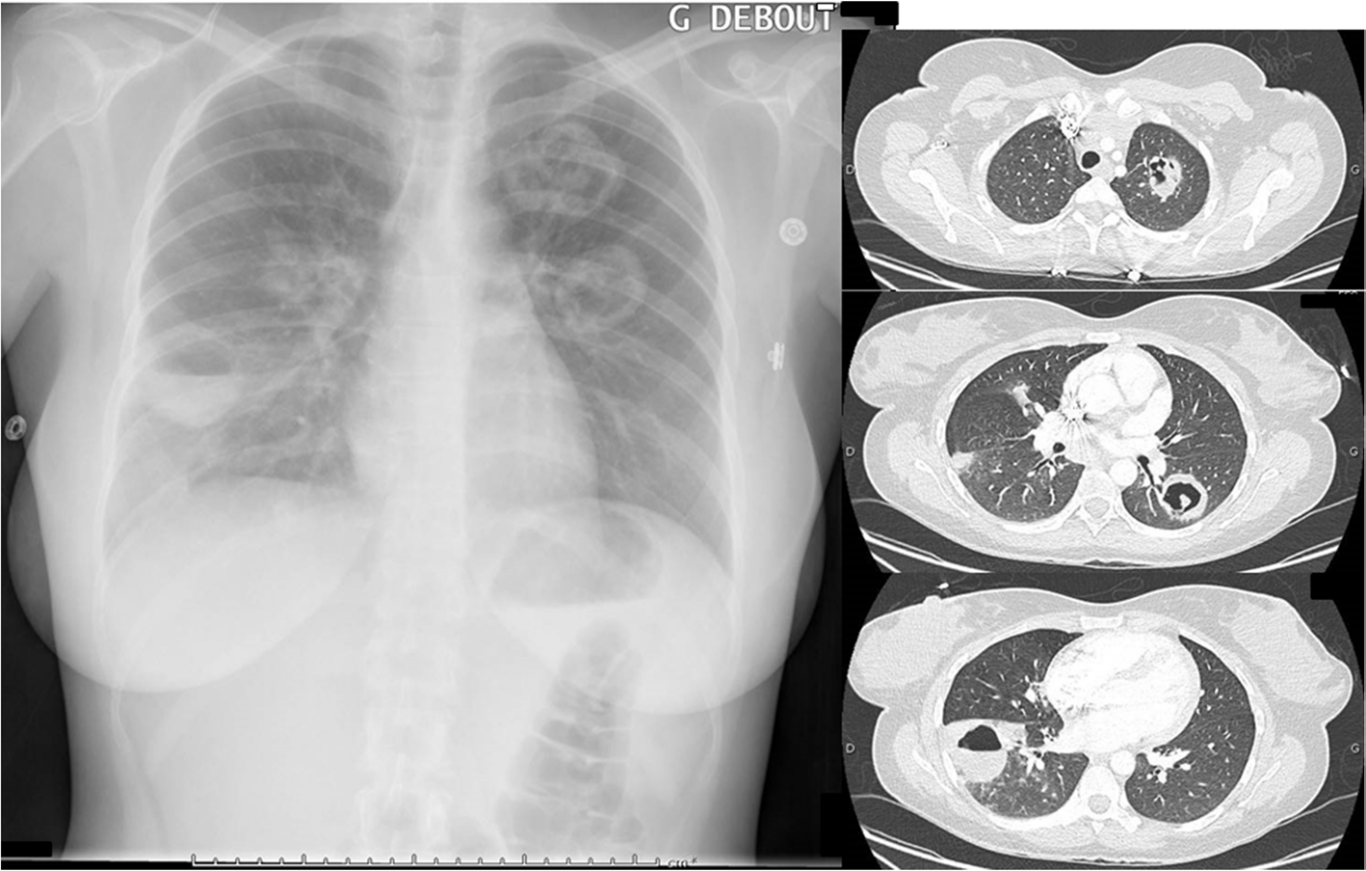
Cavitary lesions of both lungs involving upper and lower left lobes plus lower right lobe. Left: Chest X ray. Right: Corresponding Computerized tomography chest scan slices

A 25 year-old French nurse reported abrupt-onset musculoskeletal pain 15 days after delivery of her first infant. She had no past medical history and did not report any tobacco smoking, intravenous drug use or trip abroad. Her first pregnancy was uneventful except gestational diabetes. She gave birth to a healthy female neonate after an uncomplicated vaginal delivery. Fifteen days later, she reported diffuse joint pain without swelling. The pain was maximum in the morning and the joints affected were knees, ankles, wrists and shoulders. Bilateral conjunctivitis then purpuric eruption of lower limbs occurred one month after delivery. Despite a 14-day course of amoxicilline plus clavulanate (without any effect on symptoms), she was admitted to hospital because of hypochromic microcytic anaemia (7.3 g/dL; 11.5 < N < 15.1 g/dL) associated with thrombocytosis (491 000/mm3; 150 000 < N < 400 000/mm3) and elevated Serum C reactive protein (180 mg/L; N < 5 mg/L) and ferritin (560 ng/mL; 13 < N < 150 ng/mL) values. Physical examination evidenced only aphthae and small petechiae on the inner part of the leg, but no joint effusion or fever. Blood Creatinine level and proteinuria were 96 µmol/L (45 < N < 80 µmol/L) and 3000 mg/day (N < 150 mg/day) respectively. Chest X-Ray and Ct chest Scan yielded bilateral cavitary lung lesions (Fig. 1). Sputum Acid Fast Bacillus smears were negative as well as QuantiFERON®-TB Gold and blood cultures. Transthoracic Echocardiography was normal. Serum Anti -proteinase 3 antibodies level was 110UI/mL (N < 2UI/mL) and the diagnosis of Granulomatosis with Polyangiitis was retained. Outcome was favourable once methylprednisolone intravenous bolus (1000 mg/day for three days then oral prednisone 1 mg/kg/day during three weeks before progressive tapering (5 mg decrease weekly up to 30 mg, then 5 mg decrease every 2 weeks up to 15 mg, then 12 mg/day during 2 weeks, then 10 mg/day during 2 weeks before final 1 mg decrease every 2 weeks up to 5 mg/day)) and rituximab (375 mg/m^2^ weekly during four weeks followed by maintenance therapy with 500 mg every six months) were prescribed to the patient, who gave her written consent to report her case.

We made a literature review (i) to define which kinds of vasculitis were diagnosed specifically in the post-partum period (ii) to estimate how many cases of vasculitis did not have any symptoms before delivery (iii) to describe the outcome of vasculitis diagnosed in the post-partum period.

## Methods

We conducted a review of cases of vasculitis whose diagnosis was made in the post-partum period. We first screened previously published articles in Medline database using keywords “post-partum” or “puerperium” associated with “vasculitis” or “vasculitides” without time limits. Articles whose title or abstract included keywords of interest “post-partum” and/or “vasculitis” (or synonyms or causal conditions) were selected (YNG). Full text versions were obtained and only case reports or cases series with available clinical data were included; review without case reports, epidemiological works without clinical data and animal data were not included. These references were secondarily excluded (i) in case of duplicate references (ii) if the diagnosis of vasculitis was not confirmed (iii) if the diagnosis of vasculitis was made before or more than 6 months after successful delivery of a living new-born (excluding miscarriage) (iv) if vasculitis occurred only in the new born but not in the mother (v) if vasculitis related to an immunological process only affected the cerebral vasculature or the retina.

A second search in Medline using keywords “peripartum” associated with “vasculitis” or “vasculitides” without time limits was then conducted. A third search in Medline using keywords “pregnancy” associated with “vasculitis” or “vasculitides” without time limits but restricted to case reports was also conducted. A last search was conducted by checking references of previously selected references. In all these cases, the same methodology stated above to include and exclude references was applied (YNG).

**Fig. 2:**
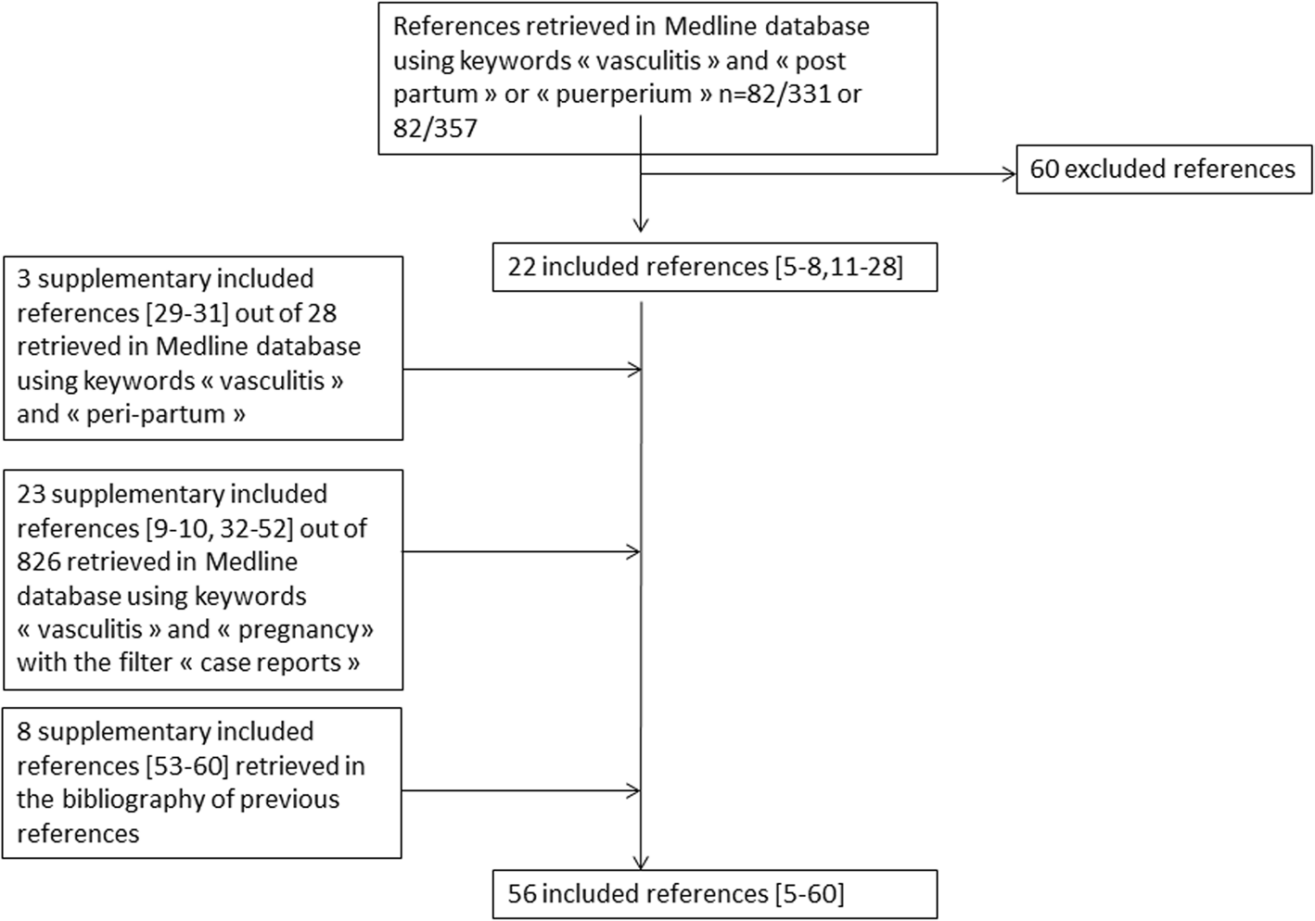
Flow chart of included references after search performed on September 20^th^, 2022

The flow chart of selected references is depicted in Fig. 2. All co-authors extracted from each case described in these references, the diagnosis of vasculitis, the symptoms and their onset as well as the pregnancy complications and the outcome. Each case was categorized in vasculitis secondary to an infectious process, vasculitis secondary to a drug exposure and vasculitis related to an immunological process (primary or secondary to connective tissue diseases, cryoglobulinemia…). The results were expressed as tables for clarity purpose and a qualitative descriptive synthesis was ultimately performed.

## Results

Fifty-six cases out of 56 references were included (Fig. 2). Among these 56 cases of vasculitis diagnosed in the post-partum period, 4 (7.1%) corresponded to vasculitis secondary to an infectious origin (Table 1) [5–8] and 2 (3.6%) corresponded to vasculitis secondary to a drug exposure (Table 2) [9, 10]. Fifty out of the 56 cases (89.3%) corresponded to vasculitis secondary to an immunological process (Table 3) [11–60]. Among these 50 cases, 44 (88.0%) corresponded to primary vasculitis, 4 were secondary to Systemic Lupus Erythematosus [26, 41, 50, 60], 1 to cryoglobulinaemia [14] and 1 to cryoglobulinaemia associated with inflammatory bowel disease [34].

**Table 1:** Vasculitis diagnosed in the post-partum period and secondary to an infectious origin

References	Year	Country	Diagnosis	Description of Symptoms associated with vasculitides	First symptom Onset	Pregnancy complications	Outcome
Thomsen et al. [5]	2015	Denmark	Cerebral vasculitis associated with Varicella Zoster Meningitis	headache, vomiting, photophobia, root pain and lesion of splenium of the corpus callosum with reduced diffusion on the diffusion-weighted sequence (MRI)	2 m p.d	none	Favourable with: -aciclovir, -corticosteroids -low dose aspirin
Subramaniam et al. [6]	2014	Malaysia	*Clostridium spp* aortitis	sudden death due to ascending thoracic aorta dissection and cardiac tamponade	7 w p.d	none	Fatal
Fabian et al. [7]	2017	Austria	Cutaneous vasculitis associated with lymph node tuberculosis	fever, axillary enlarged lymph node, papular skin rash and arthralgia of lower limb, medium size vessels vasculitis of subcutaneous fat suggestive of polyarteritis nodosa	13 w p.d	none	Favourable with: - standard antituberculous regimen - corticosteroids
Cagli et al. [8]	2010	Turkey	Pulmonary arteritis associated with mitral valve endocarditis in patient with ductus arteriosus	fever, dyspnea, fatigue, abnormal heart murmur, mobile echo-dense mass on posteromedial leaflet of mitral valve with moderate to severe mitral regurgitation, multiple vegetations in the pulmonary artery during surgery	6 w p.d	none	Favourable with: - antibiotics - open heart surgery

*MRI* Magnetic resonance Imagery, *m* month(s), *w* week(s), *p.d* post-delivery

**Table 2:** Vasculitis diagnosed in the post-partum period and secondary to a drug exposure

References	Year	Country	Diagnosis	Description of Symptoms associated with vasculitides	First symptom Onset	Pregnancy complications	Outcome
Bosnyak et al. [9]	1991	United States	Cutaneous vasculitis associated with ritodrine hydrochloride	nonpalpable, nonpruritic petechial rash	b.d	pre term labor; caesarean delivery	Favourable with: -corticosteroids
Manero-Rodriguez et al. [10]	2012	Spain	ANCA vasculitis associated with hydralazine or alphamethyldopa	leg edema; hypertension and acute renal failure	4 m p.d	HELLP syndrome; caesarean delivery	Favourable with: - corticosteroids - azathioprine

*ANCA* Anti-neutrophil cytoplasm antibodies, *m* month(s), *b.d* before delivery, *p.d* post-delivery

**Table 3:** Vasculitis diagnosed in the post-partum period and related to an immunological process

References	Year	Country	Diagnosis	Description of Symptoms associated with vasculitides	First symptom Onset	Pregnancy complications	Outcome
Berman et al. [11]	2018	United States	Microscopic polyangiitis	persistent microscopic hematuria and proteinuria; hemoptysis and bilateral infiltrates on chest radiography	b.d	chorioamnionitis; postpartum hemorrhage	Favourable with: - VVECMO - corticosteroids - plasma exchange - rituximab
Hiwarkar et al. [12]	2010	United Kingdom	Behcet’s Disease	lower limb deep vein and intracardiac thrombosis; mouth aphthous ulcers; folliculitis and livedo reticularis	10 d p.d	not specified	Favourable with: - anticoagulation - thrombolysis - corticosteroids
M’Rad et al. [13]	1989	Tunisia	Granulomatosis with Polyangiitis	fever; rhinitis, conjunctivitis; multiple opacities on chest radiography; vesicular then purpuric rash; mouth, nose and gastric ulcers; hemoptysis	2 w p.d	none	Fatal
Tocut et al. [14]	2018	Israel	Cryoglobulinaemia associated vasculitis	dyspnea; leg edema; abdominal pain; hematuria and acute renal failure; purpura and peripheral neuropathy	2 w p.d	preeclampsia	Fatal
Miyata et al.^a^ [15]	1994	Japan	Henoch-Schönlein purpura	hematuria; purpura; arthralgia and melena	b.d	none	Partially favourable with - corticosteroids
Trüeb et al. [16]	1999	Switzerland	Periarteritis Nodosa	necrotic ulceration of right breast; painful nodules of limbs sometimes leading to ulcer and acral necrosis of the left ring finger	1–3 m p.d	none	Favourable with: - corticosteroids
Lumbreras-Marquez et al. [17]	2018	Mexico	Takayasu arteritis	arterial hypertension; holosystolic murmur in the pulmonary area	b.d	chronic hypertension; caesarean delivery	Favourable with: -antihypertensive drugs
Bunker et al. [18]	2015	United States	Microscopic polyangiitis	persistent microscopic hematuria and proteinuria; dyspnea; hemoptysis; bilateral infiltrates on chest radiography and anaemia	b.d	none	Favourable with: - mechanical ventilation - corticosteroids - plasma exchange - rituximab
Singh et al. [19]	2020	India	Takayasu arteritis	arterial hypertension and differential blood pressure in both arms	b.d	chronic hypertension; preeclampsia; anhydramnios; IUGR, preterm and caesarean delivery	Favourable with: -antihypertensive drugs
Metha et al. [20]	2016	United States	Eosinophilic granulomatosis with polyangiitis	asthma; chronic sinusitis; nasal polyposis; dyspnea; left lower extremity weakness and pain; hypereosinophilia; bilateral lung consolidations with pleural and pericardial effusions on CT scan; myocarditis on MRI, cardiogenic shock, ventricular fibrillation	b.d	none	Favourable with: - dobutamine - resuscitation - implantable cardioverter-defibrillator - corticosteroids - cyclophosphamide then azathioprine
Edwards et al. [21]	2015	United Kingdom	Eosinophilic granulomatosis with polyangiitis	asthma; dyspnea; arthralgia; nodular erythematous rash; fever and rigors; nausea and abdominal pain; hypereosinophilia and alveolar shadowing plus mediastinal lymphadenopathy on CT scan	3 d p.d	not specified	Favourable with: - corticosteroids - azathioprine -cyclophosphamide then rituximab
Fason et al. [22]	2004	United States	Kawasaki Disease	fever;erythematous and pustular rash; lymphadenopathy; conjunctivitis; glossitis and cheilitis; desquamation of palm and soles	3 w p.d	none	Favourable with: - intravenous immunoglobulin
El Hajoui et al.^a^ [23]	2002	Morocco	Behcet’s Disease	arthralgia, mouth and vulvar aphthous ulcers, erythema nodosum, superior vena cava syndrome	p.d	none	Favourable with: - corticosteroids
Von Kemp et al. [24]	2019	Belgium	Eosinophilic granulomatosis with polyangiitis	asthma, sinusitis, fever; arthralgia; orthopnea; purpura hypereosinophilia; myocarditis on MRI; intracardiac thrombosis	3 w p.d	none	Favourable with: - anticoagulation - corticosteroids - cyclophosphamide then azathioprine
Mackworth Young et al. [25]	1984	United Kingdom	Granulomatosis with Polyangiitis	headache; conjunctivitis; rhinitis with midline necrosis; fever; nephrotic syndrome with pitting ankle edema; anaemia; rounded opacity infiltrates on chest radiography; splinter haemorraghe of nails; purpura and peripheral neuropathy	1 m p.d	none	Favourable with: - corticosteroids - cyclophosphamide
Hubscher et al. [26]	1984	Argentina	Pulmonary Vasculitis associated with Systemic Lupus erythomatosus	right ventricular heart failure; pulmonary embolism;	7 d p.d	proteinuria	Fatal (autoptic diagnosis
Tait et al. [27]	1955	New Zealand	Periarteritis Nodosa	arthralgia; fever; back and abdominal pain; diarrhea; pelvic abscess; hematuria; delirium; dyspnea; pericarditis; oedema; oliguria; bloody stools and hemoptysis and peripheral neuropathy	6 w b.d	none	Fatal (autoptic diagnosis)
Bolognesi et al. [28]	1955	Italy	Henoch-Schönlein purpura	arthralgia; fever; purpuric skin lesions, keratitis	1 d p.d	none	Spontaneously favourable
Damian et al. [29]	2018	Romania	Periarteritis Nodosa	fever; abdominal pain; aseptic peritonitis; ileus; ovary ischaemia; cutaneous nodules and celiac arteris stenosis	2–4 d p.d	uneventful twin pregnancy after ovarian stimulation; caesarean delivery	Favourable with: - corticosteroids - immunoglobulin therapy - cyclophosphamide then azathioprine
Nolan et al. [30]	1990	United States	Kawasaki Disease	peripartum myocardial infarction	1 d b.d	none	Not specified
Wilks et al. [31]	1993	United States	Henoch-Schönlein purpura	abdominal pain, purpuric skin lesions, arthralgia	10 d p.d	eclampsy; preterm delivery	Favourable with: - corticosteroids
Habib et al. [32]	1996	United States	Granulomatosis with Polyangiitis	sinusitis; granulomatous rhinitis; dyspnea; opacity on chest radiography; microscopic hematuria	2 y b.d	none	Favourable with: - corticosteroids - cyclophosphamide
Miller et al. [33]	1975	United States	Cutaneous leukocytoclastic angiitis	fever; myalgia; arthritis then painful cutaneous nodules^b^	4 m b.d	none	Spontaneously favourable
Hirsh et al. [34]	1980	United States	Vasculitis associated with cryoglobulinaemia and/or inflammatory bowel disease	fever; right upper quadrant pain; ascites and liver failure due to liver infarctions; pruritic purpuric rash of the lower extremities	7 d b.d	caesarean delivery for ineffective labor	Favourable with: - corticosteroids
Komaba et al. [35]	2007	Japan	Behcet’s Disease	recurrent oral aphthae; genital ulcerations; acneiform eruptions then nephrotic syndrome and extensive peripheral, iliofemoral, and caval thrombosis	b.d	not specified	Favourable with: - anticoagulation - surgical thrombectomy - corticosteroids
Aoussar et al.^a^ [36]	2007	Morocco	Takayasu arteritis	decreased brachial artery pulse; difference of > 10 mmHg in systolic blood pressure between arms; bruit over carotid, femoral and renal arteries; narrowing of carotid arteries	p.d	not specified	Favourable with: - corticosteroids
Malilolos Perez et al. [37]	1982	Spain	Periarteritis Nodosa	postpartum myocardial infarction and stroke	7 d p.d	preeclampsy; preterm delivery	Favourable with: - corticosteroids - cyclophosphamide
Stark et al. [38]^a^	1997	United Kingdom	Behcet’s Disease	recurrent orogenital ulcers and positive pathergy reaction. ^b^	3 m b.d	caesarean delivery	Not specified
Giles et al. [39]	1986	Australia	Takayasu arteritis	systolic ejection murmur at the left sternal edge; weak femoral pulses; stenosis of abdominal aorta, renal and mesenteric arteries	b.d	chronic hypertension; preterm and caesarean delivery	Not specified
Umeda et al. [40]	2003	Japan	Takayasu arteritis	incidental finding of abdominal aorta aneurysm during pregnancy	b.d	none	Not specified
Rubin et al. [41]	1994	Canada	Pulmonary Vasculitis associated with Systemic Lupus erythomatosus	dyspnea; right Ventricular Heart Failure	2 d p.d	chronic hypertension; preeclampsy; preterm and caesarean delivery	Fatal (autoptic diagnosis)
Diamanti et al. [42]	2014	Italy	Eosinophilic granulomatosis with polyangiitis	asthma; rhinitis; sinusitis; mononeuritis multiplex; hypereosinophilia and cervical spinal intradural haemorrhage	b.d	not specified	Favourable with: - corticosteroids - rituximab
Nicolas et al. [43]	2005	France	Takayasu arteritis	abdominal pain, headache; transient brachiofacial paralysis; hypertension; dyspnea; decreased brachial artery pulse; difference of > 10 mmHg in systolic blood pressure between arms; bruit over carotid and stenosis of carotid and subclavian arteries	2 y b.d	not specified	Favourable with: - corticosteroids
Ogasawara et al. [44]	1995	Japan	Eosinophilic granulomatosis with polyangiitis	asthma; fever; hypereosinophilia; pulmonary infiltrates and mononeuritis	3 m p.d	none	Favourable with: - corticosteroids
Bessias et al. [45]	2005	Greece	Granulomatosis with Polyangiitis	hemoptysis; cough; right leg pain; bilateral parenchymal infiltrates on chest radiography and limb ischaemia due to recurrent arterial thrombosis	b.d	preterm and caesarean delivery	Favourable with: - corticosteroids - cyclophosphamide - leg amputation
Corradi et al. [46]	2009	Italy	Eosinophilic granulomatosis with polyangiitis	asthma; fever; polyarthralgia; chest pain; skin petechiae; left ventricular dysfunction; bilateral alveolointerstitial infiltrates on chest radiography; hypereosinophilia	2–8 w p.d	none	Favourable with: - corticosteroids - left ventricular heart assistance device - heart transplantation
Sahni et al. [47]	2005	India	Granulomatosis with Polyangiitis	cough; fever; rhinitis; multiple nodular parenchymal opacities on chest radiography; sinusitis	2 m b.d	none	Favourable with: - corticosteroids - cyclophosphamide
Casellas et al. [48]	1993	Spain	Buerger’s disease	raynaud's disease with distal necrosis of the middle finger of the right hand; absence of left radial and pedal pulse; multiple stenoses and screwdriver lesions in radial, cubital, and palmar arches	9 y b.d	IUGR, preterm and caesarean delivery	Not specified
Gasch et al. [49]	2009	Spain	Takayasu arteritis	fever; aneurysms of abdominal aorta and left subclavian artery; stenosis of carotid arteries	10 d p.d	uneventful twin pregnancy	Favourable with: - corticosteroids - abdominal aneurysm surgery
Borahay et al. [50]	2009	United States	Cutaneous vasculitis associated with Systemic Lupus erythomatosus	fever; purpuric patchy lesion of hands and feet	1 d p.d	eclampsy; caesarean delivery	Favourable with: - corticosteroids - hydroxychloroquine
Lima et al. [51]	1995	United Kingdom	Granulomatosis with Polyangiitis	Sinusitis; sore throat; arthralgia; itchy ears; fever; sweats	6 w p.d	not specified	Favourable with: - corticosteroids - azathioprine
Bharuthram et al. [52]	2020	South Africa	Takayasu arteritis	bruit over carotid; right carotid and both femoral arteries stenosis and thoracic aorta and right subclavian artery dilatations	b.d	preterm delivery	Favourable with: - corticosteroids - azathioprine
Cooper et al. [53]	1970	United Kingdom	Granulomatosis with Polyangiitis	epistaxis; rhinitis; deafness, facial palsy; cavitary lesion on chest radiography	1 m p.d	not specified	Favourable with: - corticosteroids - azathioprine
Dey et al. [54]	2015	India	Takayasu arteritis	arterial hypertension; upper limb pulselessness with unrecordable blood pressure; carotid and subclavian arteries stenosis	6 m b.d	none	Not Specified
Hiyama et al. [55]	2000	Japan	Eosinophilic granulomatosis with polyangiitis	asthma; fever; abdominal pain; diarrhea; hypereosinophilia; bilateral micronodular pulmonary infiltrates and mononeuritis multiplex	4 w p.d	none	Favourable with: - corticosteroids
Abdul-Haj et al. [56]	1961	United States	Eosinophilic granulomatosis with polyangiitis	fever; cough; asthma, pleural and pericardial effusion; dyspnea and hypereosinophilia	2 y b.d	none	Fatal (autoptic diagnosis)
Uchida et al. [57]	1994	Japan	Eosinophilic granulomatosis with polyangiitis	rhinitis; asthma; bilateral pulmonary infiltrates; eosinophilia; mononeuritis multiplex; abdominal pain and diarrhea	b.d	not specified	Favourable with: - corticosteroids
Siegler et al. [58]	1965	United States	Periarteritis Nodosa	seizure; acute renal failure then intestinal bleeding	b.d	eclampsy; preterm and caesarean delivery	Fatal (autoptic diagnosis)
Bassa et al. [59]	1995	South Africa	Takayasu arteritis	claudication of upper limbs; brachial and radial pulses weak or absent; decreased blood pressure of upper limb and ectacic and stenotic lesions of both carotid arteries	4 m p.d	caesarean delivery	Favourable with: - corticosteroids
Suzuki et al. [60]	1990	Japan	Leptomeningeal vessels vasculitis associated with Systemic Lupus erythomatosus	fever; headache, stiffneck; seizure; oculomotor nerve palsy; ataxia; paraplegia; respiratory arrest and autoptic findings of necrotizing vasculitis of small vessels of heart, liver, pancreas, gall and urinary bladders	11 d b.d	eclampsy; preterm and caesarean delivery	Fatal (autoptic diagnosis)
This case	2022	France	Granulomatosis with Polyangiitis	musculoskeletal pain involving knees, ankles, wrists and shoulders; bilateral conjunctivitis; purpuric eruption of lower limbs; aphtae; proteinuria and bilateral cavitary lung lesions	2 w p.d	gestational diabetes	Favourable with: - corticosteroids - rituximab

*CT scan* computerized tomography scan, *MRI* Magnetic resonance Imagery, *VVECMO* venovenous extracorporeal membrane oxygenation, *y* year(s), *m* month(s), *w* week(s), *d* day(s), *b.d* before delivery, *p.d* post-delivery, *IUGR* intra-uterine Growth Restriction

^a^ uncertain timeline

^b^associated newborn vasculitis

The primary vasculitis diagnosed were Takayasu Arteritis (*n* = 10) [17, 19, 36, 39, 40, 43, 49, 52, 54, 59], Eosinophilic granulomatosis with polyangiitis (n = 9) [20, 21, 24, 42, 44, 46, 55–57], Granulomatosis with Polyangiitis (*n* = 7) [13, 25, 32, 45, 47, 51, 53], Periarteritis Nodosa (*n* = 5) [16, 27, 29, 37, 58], Behcet’s disease (*n* = 4) [12, 23, 35, 38], Henoch-Schönlein purpura (*n* = 3) [15, 28, 31], Microscopic polyangiitis and Kawasaki Disease (two cases each) [11, 18] and [22, 30], Buerger’s disease and Cutaneous leukocytoclastic angiitis (one case each) [48] and [33].

Among the 44 primary vasculitis diagnosed in the post-partum period, the first symptom occurred before delivery in 26 cases (59.1%) [11, 15, 17–20, 27, 30, 32–35, 38–40, 42, 43, 45, 47, 48, 52, 54, 56–58, 60]. Among the 6 vasculitis secondary to Systemic Lupus Erythematosus and cryoglobulinaemia [14, 26, 34, 41, 50, 60], the first symptom of vasculitis occurred before delivery in 2 cases only (33.3%) [34, 60]. There was no pregnancy complications in 11 out of the 28 (39.2%) vasculitis cases secondary to an immunological process (primary and secondary) whose first symptom occurred before delivery and in 11 out of 22 (50.0%) vasculitis cases secondary to an immunological process (primary and secondary) whose first symptom occurred after delivery. Maternal death occurred in 4 out of the 44 primary vasculitis (9.1%) [13, 27, 56, 58] and in 4 out of the 6 vasculitis (66.6%) secondary to Systemic Lupus Erythematosus and cryoglobulinaemia [14, 26, 41, 60]. Two infants born to mothers with vasculitis symptoms before delivery experienced vasculitis a few days after birth [33, 38].

## Discussion

In this review, the systemic vasculitis diagnosed in the post-partum period (i.e. during the first 6 months following delivery) were mainly due to an immunological process and corresponded to primary vasculitis in more than 75% of cases. All kinds of vasculitis have been described (large, medium and small vessels) but the five most common diagnoses were Takayasu Arteritis, Eosinophilic granulomatosis with polyangiitis, Granulomatosis with Polyangiitis, Periarteritis Nodosa and Behcet’s Disease. In less than half of cases, there were no symptoms reported before delivery, but in the other half, reported symptoms did not lead to a diagnosis before delivery and to the exclusion from this review (see exclusion criteria above). The maternal mortality of the primary vasculitis diagnosed in the post-partum period was less than 10%, but the maternal mortality of the vasculitis secondary to Systemic Lupus Erythematosus and cryoglobulinaemia appeared higher, within limitations of a low number of cases.

Among primary vasculitis (which accounted for the majority of vasculitis diagnosed in the post-partum period), the distribution of the diagnosis was close to that observed during pregnancy [4], suggesting that vasculitis diagnosed in the post-partum were the continuity of those diagnosed during pregnancy and therefore belonged to the same nosological entity. Indeed, most authors of such cases considered post-partum as a part of pregnancy because more cases were retrieved with the keyword “pregnancy” than with the keyword “post-partum” or “puerperium” (Fig. 2).

The most frequently described vasculitis in our review and in that of Gatto and colleagues focusing on pregnancy [4] was Takayasu Arteritis. It was the sole vasculitis involving large arteries described because, unlike giant cell arteritis, it occurred especially among women of childbearing age. Moreover, Takayasu Arteritis is likely to be overrepresented in this review focusing on vasculitis diagnosed within 6 months post-delivery, because angiographic investigations required to confirm the diagnosis were performed after delivery in all cases [17, 19, 36, 39, 40, 43, 49, 52, 54, 59]. The risk of intra-uterine growth restriction, the need of close monitoring of Blood Pressure and the anaesthetic management of Takayasu Arteritis during labour have been reviewed elsewhere [3, 61–64].

Periarteritis nodosa was the main vasculitis involving medium arteries in our review and in that of Gatto [4]. The main vasculitis involving small arteries were Eosinophilic granulomatosis with polyangiitis and Granulomatosis with Polyangiitis, 2^nd^ and 3^rd^ most common vasculitis in our review versus 4^th^ and 3^rd^ in the review of Gatto and colleagues [4]. Why Behcet’s disease (vasculitis involving variable vessels including veins [2]) was underrepresented in our review (5^th^ more common diagnosis versus 2^nd^ in the review of Gatto and colleagues [4]) remained misunderstood. Maybe it could be due to a reporting bias and to undiagnosed Behcet’s diseases in the post-partum when considering genital ulcerations or deep vein thrombosis as genuine complications of delivery.

Within limitations of a low number of cases, the vasculitis secondary to Systemic Lupus Erythematosus and cryoglobulinaemia seemed to have a higher occurrence of symptoms after delivery and a higher mortality than primary vasculitis. In those latter, the diagnosis was not made before delivery even if the first symptoms occurred before delivery in 59.1% of cases. The occurrence of neonatal vasculitis was only observed in two infants born to mothers with vasculitis symptoms before delivery [33, 38]. All these assertions suggested that the physicians should pay attention to symptoms and keep a high degree of suspicion for vasculitis before as well as after delivery. We agreed that we should remain cautious when analysing mortality from these cases included during a large time period with different practices for diagnosis as well as for therapeutic purposes.

The main limitation of this review remains its non-exhaustive nature. Even if we try to build a systematic reproducible review, some relevant references were found out of the scope of the search we made [65]. Conversely, we were unable to find some full texts [66, 67]. Moreover, we exclude references with unconfirmed diagnosis [68], very uncertain timelines [69] or diagnosis made more than 6 months after delivery [70], because 6 months is considered as the maximal length of the post-partum period for some authors [71] and as the time delay during which the hormonal environment leading to ovulation has not been restored in more than 80% of breastfeeding women of Western countries [72]. The cases of vasculitis affecting only the neonate [73] were out of the scope of this review, as well as the cases of vasculitis related to an immunological process involving only the cerebral vasculature or the retina [74, 75]. In these cases, other pathological processes such as vasospasm could not be ruled out as a differential diagnosis of vasculitis [76–78]. Although non-exhaustive, we tried to make this review as systematic as possible.

In conclusion, the data from this review suggested that the vasculitis diagnosed in the post-partum period were mainly primary vasculitis among patients in whom the diagnosis has not been made during pregnancy. In less than half of cases and as described in ours, there were no reported symptoms before delivery. Therefore, the physicians should pay attention to symptoms and keep a high degree of suspicion for vasculitis before as well as after delivery.

## Data Availability

Data sharing is not applicable to this article as no new data were created or analyzed in this study.
